# High-Performance Nylon-6 Sustainable Filaments for Additive Manufacturing

**DOI:** 10.3390/ma12233955

**Published:** 2019-11-28

**Authors:** Ilenia Farina, Narinder Singh, Francesco Colangelo, Raimondo Luciano, Giulio Bonazzi, Fernando Fraternali

**Affiliations:** 1 Department of Engineering, University of Naples “Parthenope”, Centro Direzionale di Napoli, Isola C4, 80143 Naples, Italy; ilenia.farina@uniparthenope.it (I.F.); francesco.colangelo@uniparthenope.it (F.C.); raimondo.luciano@uniparthenope.it (R.L.); 2 Department of Civil Engineering, University of Salerno, Via Giovanni Paolo II 132, 84084 Fisciano, SA, Italy; snarinder@unisa.it; 3 Aquafil s.p.a., via Linfano 9, 38062 Arco (Trento), Italy; giulio.bonazzi@aquafil.com

**Keywords:** nylon-6, additive manufacturing, recycling, melt flow index, tensile strength, wear resistance, warping

## Abstract

This study deals with the development of Nylon-6 fused deposition modeling (FDM) filaments for additive manufacturing, which couples high mechanical performances with eco-sustainability. These filaments were extruded from recycled Nylon-6 granulates through a dedicated twin-screw extrusion line, which processes either pure Nylon-6 grains, or mixtures of such a material with minor fractions of acrylonitrile butadiene styrene (ABS) and titanium dioxide (TiO_2_). The rheological and thermal properties of the investigated filaments are analyzed, including melt flow index, melting temperature, and decomposition temperature, which are of the utmost importance when avoiding the overheating and decomposition of the material. Such a study is conducted in both pre-extrusion and post-extrusion conditions. The tensile strength, the wear resistance, and the printability of the examined recycled Nylon-6 filaments are also studied by comparing the properties of such filaments with those exhibited by different nylon-based filaments for FDM that are available in the market. The given results show that the recycling of Nylon-6 through the “caprolactam” regeneration route enables the newly formed material to retain high physical and mechanical properties, such as tensile strength at yield in the interval 55.79–86.91 MPa. Referring to the basic composition of the filaments examined in the present study, this remarkably high-yield strength is accompanied by a Young modulus of 1.64 GPa, and wear resistance of 92 µm, under a 15 min/1 kg load pin-on-disk test carried at the sliding speed of 250 rpm.

## 1. Introduction 

There is growing scientific interest in the recycling of plastic solid waste (PSW) to form composite materials with enhanced physical–mechanical properties [1,2,3,4,5]. The reuse of recycled materials is a necessity today because of the difficulty of finding new places for landfilling the generated waste from various sources [4]. Various recycling and recovery techniques have been significantly improved over recent years, with the aim of optimizing the reuse of PSW [4,5], and recycling through extrusion processes has received particular attention [6,7,8,9,10,11]. The recycling of plastic materials is highly helpful in reducing the harmful impact of PSW on the environment; and this is particularly true in the case of nylon waste, since nonbiodegradable nylon products, such as fishing nets, are often indiscriminately abandoned in the oceans [12]. It is worth mentioning that nylon has good fluidity and is widely used to produce mono filaments, hinges, ropes, and profiles. It is a semi-crystal polyamide polymer that has a very low specific weight (1.14 g/cm^3^), excellent tensile strength (31–80 MPa), toughness, high break strain (20% to 60%), excellent elastic recovery, and resistance to bending and wear [13,14]. Since the discovery of nylon, several efforts have been made to develop such a material further for various high-end applications because of its commendable properties. In the area of additive manufacturing, increasing attention is being paid to the use of different types of nylon, to form 3D printing filaments (e.g., [15] and references therein). It is worth noting that recycled nylon granulates, to be directed to extrusion processes, can be effectively obtained through a variety of recycling processes [15,16,17].

A very efficient regeneration and recycling process of Nylon-6 waste collected from various resources has been developed by the internationally renowned Aquafil group through the ECONYL^®^ project [18]. This group has developed a three-step system to produce recycled Nylon-6 (R-Nylon-6) from 100% regenerated waste materials, which includes fishing nets abandoned in the oceans and aquaculture, as well as scraps from carpets and various industrial nylon products otherwise directed to landfills. The first step of the ECONYL^®^ process consists of the collection of Nylon-6 waste from landfills, oceans, and all over the world. Step two develops an accurate regeneration and purification process, articulated in the depolymerization of the collected Nylon-6 waste (with transformation of the recycled material into caprolactam), purification of caprolactam, and re-polymerization [18]. Such a process drives the material back to its original purity, obtaining a regenerated material (hereafter simply referred to as AQ R-Nylon-6, where the acronym AQ is introduced to refer to the commercial R-Nylon-6 products AQ24000, AQ27000, and AQ34000 by Aquafil), which exhibits physical and mechanical properties practically identical to those of the virgin material [18]. Step three consists of the manufacturing of AQ R-Nylon-6 granulates to be processed for the fabrication of carpet and textile yarns or filaments, to be employed for a variety of industrial uses which do not currently include the manufacturing of Fused Deposition Modeling (FDM) filaments.

The high mechanical performances of AQ R-Nylon-6, and the possibility to make use of granulates obtained through the accurate ECONYL^®^ regeneration process, motivated us to focus the present study on a comprehensive investigation into the physical, mechanical, and wear-resistance properties of AQ R-Nylon-6 filaments for FDM. The extrusion of AQ R-Nylon grains, alone or combined with minor fractions of Acrylonitrile Butadiene Styrene (ABS) and titanium dioxide (TiO_2_), has been performed through a dedicated pilot line located in the Aquafil plant of Arco (Trento, Italy), as described in Section 2.2. The addition of ABS grains was considered in order to study its influence on the reduction of the Melt Flow Index (MFI) of the composite filaments, while that of TiO_2_ particles was analyzed with the aim of conferring a white color to the filaments, which leads to a uniform and clean finish of the 3D-printed objects. The properties of the AQ R-Nylon-6 filaments for FDM are analyzed by drawing comparisons with the analogous properties of FDM filaments manufactured from non-regenerated R-Nylon-6 (NR R-Nylon-6), which were investigated in the technical literature over recent years [19,20,21,22,23,24], and some commercially available filaments made from virgin nylon materials. The latter include Nylon-Polyamide 6 (PA6) Low Warp by Spectrum [25], Nylon-645 [26], and Nylon-680 [27] by Taulman3D, as well as Nylon-11 and Nylon-12 filaments for 3D printing [28,29]. The results presented in this paper demonstrate that the analyzed filaments made from regenerated Nylon-6 waste show tensile yield strength varying from 55.79 MPa up to 86.91 MPa, which is significantly higher than that exhibited by the comparative commercial filaments (% increments even higher than 100%); Young modulus in the interval 0.76–2.34 MPa; considerably high elongation at break (≈ 40%); thermal stability, noticeable wear resistance and good printability.

## 2. Experimental

### 2.1. Materials

**Figure 1:**
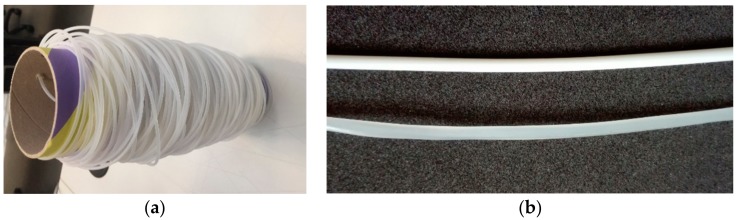
Pictures illustrating the AQ27000 filaments: (**a**) manufactured in the Aquafil plant of Arco (Trento, Italy) and a comparison between an AQ24000-T filament (**b**, top) and an AQ27000 filament (**b**, bottom).

Five different AQ R-Nylon-6 filaments were tested, and some of them were prepared by mixing AQ R-Nylon-6 grains with reinforcements such as ABS and TiO_2_. The Nylon-6 grains have different viscosity numbers, which are expressed in milliliters per gram (mL/g), and are measured according to ISO 307/2007 “Plastics-Polyamides-Determination of viscosity number” [30], through a dilute solution of PA6 and sulfuric acid. The examined filaments are 1.75 mm in diameter and correspond to the following types:

- AQ27000 R-Nylon-6 filaments with viscosity number 2.7 mL/g (polymer “Aquamid AQ27000” by Aquafil, hereafter referred to as AQ27000 filaments, see Figure 1a and bottom of Figure 1b);
- AQ34000 R-Nylon-6 filaments with viscosity number 3.4 mL/g (polymer “Aquamid AQ34000”, hereafter referred to as AQ34000 filaments);
- AQ27000 R-Nylon-6–ABS 5% polymer filaments made up of 95% polymer weight AQ27000 and 5% in ABS SM295 (Injection Molding Grade) polymer weight by LG Chemical Ltd. (Seoul, Korea) (AQ27000-B filaments);
- AQ34000 R-Nylon-6–ABS 5% filaments made up of 95% polymer weight AQ34000 and 5% in ABS SM295 polymer weight from LG Chemical Ltd. [31] (AQ34000-B filaments);
- AQ24000 R-Nylon-6–TiO_2_ 30% filaments (top of Figure 1b) consisting of 70% weight polymer “Aquamid AQ24000” (viscosity number 2.4 mL/g) and 30% in weight from titanium dioxide TiO_2_ made available by Aquafil (AQ24000-T filaments) (Arco, TN, Italy).

The following properties were declared by the supplier LG Chem for the employed ABS: specific gravity between 1.01 and 1.04; melt flow rate equal to 10 g/10 min; tensile strength of 34.3 MPa; break elongation of no less than 10%; Young’s modulus of 1.578 GPa [31].

In addition to the AQ R-Nylon-6 filaments, the NR R-Nylon-6 filaments studied by Singh et al. [19,20,21,22,23,24] were analyzed. Such filaments employ a non-regenerated recycled Nylon-6 as matrix material, which is either used alone [19] or in association with SiC (silicon carbide) and Al_2_O_3_ (aluminum oxide) reinforcements [20,21,22,23,24]. For this experimentation work, recycled Nylon-6 granules that were not subject to a regeneration treatment, as in the case of AQ R-Nylon-6 [18], were acquired from the Gujarat fertilizers limited (Vadodara, India) [32].

### 2.2. Manufacturing Process

Since nylon has little biodegradability in the atmosphere, it is an ideal candidate for recycling processes [33,34]. Various extrusion processes are available to produce filaments to be used as a raw material in 3D printing processes of the FDM. Single-screw extrusion is a conventional process to produce filaments; however, it may produce manufacturing defects, small pores, air bubbles, and nonuniform mixing [33,34,35,36]. Twin-screw extrusion has emerged as an advanced technique to produce filaments in which such defects are negligible or absent, since this extrusion process allows a high and uniform dispersion of reinforcing particles in the matrix material [20,21,22,23,24,36,37]. The AQ R-Nylon-6 filaments examined in the present study were manufactured through a dedicated extrusion line in the Aquafil plant of Arco (Trento, Italy), equipped with a co-rotating twin-screw extruder, type Scientific LTE 20–40 (LTE Scientific Ltd, Greenfield, Oldham, UK), featuring a length (L) to diameter (D) ratio of 40:1, 21 mm diameter screws, 10 housing sections, and dye made of stainless steel for 1.75 mm diameter filament making (operation speed: 600 rpm). The following thermal profile was employed across the ten different zones of the screw: zone adjacent to the hopper: 110 °C; central zones 2–9: 250 °C; zone 10 adjacent to the dye: 260 °C.

## 3. Characterization 

### 3.1. Melt-Flow-Index Evaluation Tests

The melt flow index (MFI) is the amount, expressed in grams, of molten plastic material being passed in 10 min from a capillary under the thrust of a certain weight and at a certain temperature, according to the ASTM D1238 standards [38,39]. In this work, we characterize such a property by using a melt-flow-index tester featuring the following technical specifications: weight: 2.16 kg; maximum temperature range: ambient to 300 °C; resolution: 0.10 °C; accuracy: + 0.20 °C; timer: 0–99.9 min. The MFI instead provides rough information on the fluid dynamic behavior of the polymer in test conditions [31], which can serve to determine the plastic behavior of the material under high temperatures.

The following sections provide MFI test results for NR and AQ R-Nylon-6, which are aimed at investigating the changes in material flow behavior before and after the extrusion process. The presented results were obtained according to ASTM D1238, by using the melt-flow-index tester able to work up to 300 °C, which is accurately described in [19].

### 3.2. Melt-Flow-Index Evaluation Tests for NR R-Nylon-6

**Figure 2:**
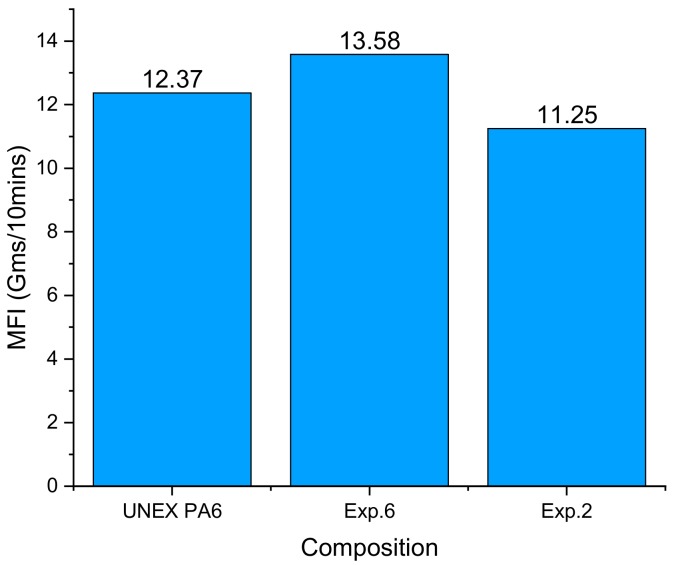
Pre- and post-extrusion MFI comparison for pure NR R-Nylon 6.

Figure 2 shows the results of the MFI tests presented in [19] for NR R-Nylon-6 with reference to a pre-extrusion material sample (Un-Extruded PA6/UNEX PA6), and two different post-extrusion samples (Exp 2 and Exp 6). The latter correspond to the extrusion experiments presented in [19], which were conducted to manufacture the NR R-Nylon-6 filaments with the extreme (maximum/minimum) mechanical properties (cf. Section 3.5.1). The results in Figure 2 show that the highest MFI (11.38 g/10 min) is exhibited by the post-extrusion sample Exp 6.

Boparai et al. also analyzed the MFI of three different blends of NR R-Nylon-6, Al and Al_2_O_3_ (results in Table 1), which were aimed at making 1.75 mm diameter filaments enriched with reinforcing particles of different sizes [20,21,22,23,24]. Moving from the composition named with the acronym A to the composition named with the acronym C in Table 1, the weight percentage of NR R-Nylon-6 remains constant, while that of Al grows, and, at the same time, that of Al_2_O_3_ decreases gradually (2%).

The results in Table 1 highlight that the addition of Al and/or Al_2_O_3_ particles to the NR R-Nylon-6 filaments lead to greatly decreases the MFI from ≈12 g/10 min (unreinforced material) to ≈2.20 g/10 min (post-extrusion tests). This happened due to the increase in the overall density of the blend after blending of ceramic reinforcements, such as Al and Al_2_O_3_. It is worth noting that the MFI exhibited by NR-R-Nylon-6/Al_2_O_3_ composite filaments is close to that of commercial ABS filaments for FDM, which is 2.41 g/10 min [23].

### 3.3. MFI Tests for AQ R-Nylon-6

**Figure 3:**
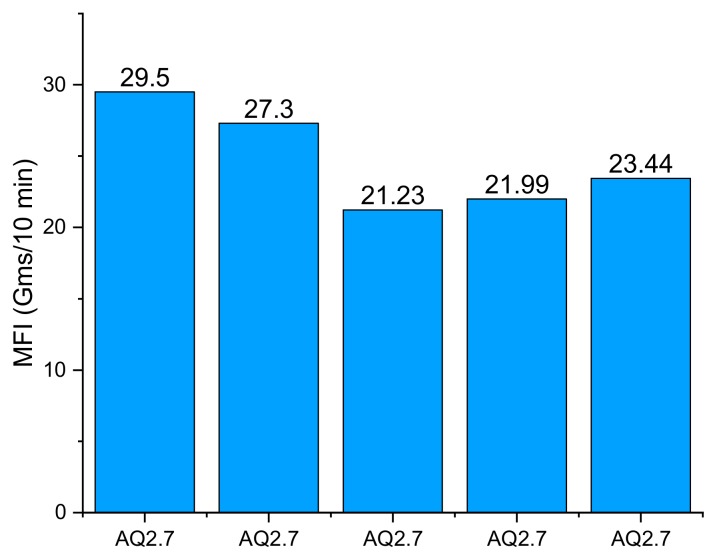
MFI performance of AQ27000 filaments on five repetitions (AQ2.7 stands for AQ27000).

This section illustrates the MFI tests performed on the AQ R-Nylon-6 filaments prepared by Aquafil through the dedicated twin-screw extrusion line described in Section 2.2, Table 2 and Figure 3 show the results of the MFI tests performed on AQ27000 filaments in five repetitions, along with the average recorded value (Avg column). Table 3 instead shows the deviations between the average MFI values exhibited by the AQ27000-B, AQ34000, AQ34000-B, and AQ27000-T 30% filaments and the average value of the MFI exhibited by the AQ27000 filaments. The addition of ABS to AQ filaments has been considered because of the easy extrusion of ABS and the ABS filaments are among the strongest available polymer filament filaments for 3D printing. Additionally, TiO_2_ has been added to improve mechanical properties of basic AQ R-Nylon-6, and to confer a uniform white color to the filaments (cf. top of Figure 1b) [40].

From the analysis of the results shown in Table 2 and in Table 3, it is clear that AQ27000 filaments exhibit significantly larger MFI than all the other AQ R-Nylon-6 filaments analyzed in this study, except for the AQ27000-T composite filaments. The average MFI of the AQ27000 filaments is 24.69 g/10 min and is about 10 times higher than that exhibited by ABS filaments commonly used in FDM processes (2.4 g/10 min) [19]. It should be noted that the AQ27000-B filaments exhibit the lowest fluidity index among the fibers examined (19.24 g/10 min), which is in fact slightly lower than the MFI exhibited by both the AQ34000 (21.71 g/10 min) and the AQ34000-B filaments (20.26 g/10 min). We will see in Section 3.5, Section 3.6 and Section 3.7 that the high fluidity of the AQ filaments, much greater than the reference ABS value, does not affect their good mechanical performances and their printability.

### 3.4. Thermal Characterization of R-Nylon-6 Granulates (DSC)

#### 3.4.1. Pre- and Post-Extrusion DSC Test for NR R-Nylon-6

The influence of the process temperature on the properties of the extruded material can be conveniently studied by comparing the results of Differential Scanning Calorimetry (DSC) tests in pre- and post-extrusion conditions. The results of DSC tests on NR R-Nylon-6 granules, before extrusion, were obtained by Singh et al. in [19] through three cycles of heating and cooling. It was observed that the melting point of NR R-Nylon is 220 °C in the first heating cycle and equal to 219.4 °C and 218.91 °C in the second and third cycles, respectively [19]. Based on the observations of the DSC tests, it can be concluded that processing through NR R-Nylon-6 melting processes can be performed up to 250–260 °C, which is in line with the process values typically adopted by Aquafil (cf. Section 3.4.2).

DSC tests were also performed on the NR R-Nylon-6 filaments corresponding to the extrusion experiments No. 6 and No. 2 described in [19]. The results of the tests illustrated in such a reference show that extrusion has significant beneficial effects on material melting point behavior. As a matter of fact, the trial conducted in three continuous cycles of heating and cooling highlighted that the extrusion leads to an increase in the melting temperature and the solidification temperature. These variations are visible in the DSC curves presented in [19] and suggest that non-extruded NR R-Nylon-6 granules are less stable from a melting and solidification perspective, while extruded NR R-Nylon-6 is more thermally stable.

#### 3.4.2. Pre- and Post-Extrusion DSC Test for AQ R-Nylon-6

**Figure 4:**
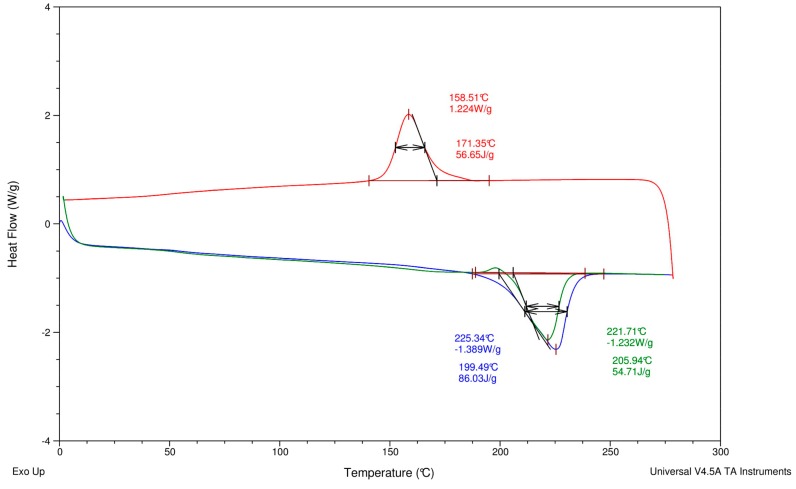
Pre-extrusion DSC test on AQ27000 granules.

**Figure 5:**
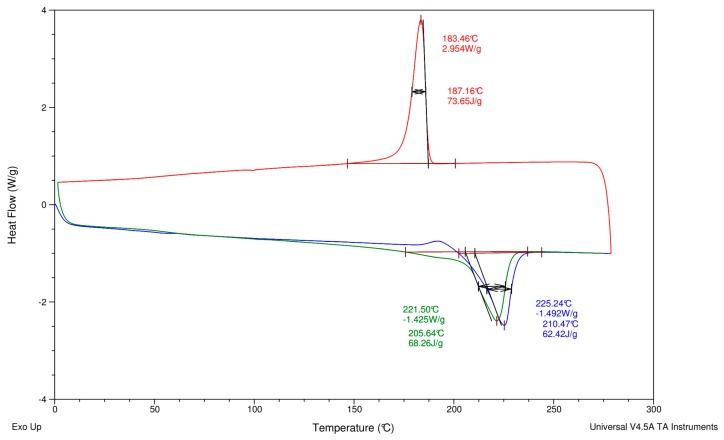
Post-extrusion DSC test on AQ27000 filaments.

DSC tests were performed on the AQ27000 granules and filaments produced by extrusion at the Aquafil plant of Arco (Trento, Italy), through a Heat/Cool/Heat method on a DSC Q20 V24.4 Build 116 module produced by TA Instruments. No postprocessing of the material was carried out after extrusion. The results of the DSC tests represented in Figure 4 (pre-extrusion conditions, sample weight 9.4 mg) and Figure 5 (post-extrusion conditions, sample weight 7.0 mg) highlight that the extrusion process does not markedly change the melting temperature of the AQ27000 material; the results also reveal the remarkable thermal stability of such a material when subjected to heating and cooling cycles.

After carefully observing Figure 4 and Figure 5, one observes that the melt-processing temperature of the material does not markedly change in pre- and post-extrusion conditions. A small change is observed, which is, however, not greatly relevant, since, in extrusion processes, small amplitude changes in the melting temperature can often be observed, and this does not significantly alter the thermal properties of the material. It can therefore be concluded that, after recycling and regenerating Nylon-6 waste through the ECONYL^®^ process [18], negligible changes in processing conditions and thermal properties can be observed in the material before and after extrusion, which suggests that such a recycling process is well suited for the FDM application domain.

When comparing DSC test results on AQ R-Nylon-6 and NR R-Nylon-6 filaments, one further observes that the melting temperature of the AQ R-Nylon-6 filaments in pre and post-extrusion stages remains in the 221 to 225 °C range, while the melting temperature of the NR R-Nylon-6 filaments stays in the 220 to 225 °C range [21]. This proves that AQ R-Nylon-6 can be extruded by using process parameters similar to those used for NR R-Nylon-6 [19], as we already observed.

### 3.5. Tensile Tests

#### 3.5.1. Tensile Tests for NR R-Nylon-6

The results of tensile tests on NR R-Nylon-6 filaments were presented in [19,20,21,22,23,24], after extrusion from a laboratory single-screw line. Nine different extrusion experiments were carried out by tuning the extrusion parameters and tensile tests through a Taguchi L9 array approach. Use was made of a universal tensile tester by Shanta Engineering, Thane, India, with maximum capacity 10 kN, maximum crosshead displacement 900 mm, and column clearance 380 mm, operating according to the ASTM D638 standard. This study was aimed at optimizing the extrusion process parameters of pure and blended NR R-Nylon-6 filaments. The optimized pure NR R-Nylon-6 filaments studied in [19] showed 9.02 MPa yield strength; 0.28 GPa Young modulus; and 14% elongation at break. The composite filaments corresponding to the A, B, and C blends listed in Table 1 exhibited 21.40 MPa (A), 21.53 MPa (B), and 21.65 MPa (C) tensile strengths; 0.58 GPa (A), 0.76 GPa (B), and 1.17 GPa (C) Young moduli; and 18.62% (A), 12.74% (B) and 8.56% (C) elongations at break. The results presented in [20,21,22,23,24] show that the Young modulus of the Al/Al_2_O_3_ composite filaments with NR R-Nylon-6 matrix is significantly lower than that of the ABS filaments examined for comparison (1.63 GPa), while the tensile strength of the former is only slightly lower than that of the ABS filaments (22.00 MPa). This indicates that Nylon-6/Al/Al_2_O_3_ filaments are competitive with commercial ABS filaments, being characterized by a comparable mechanical strength.

#### 3.5.2. Tensile Test for AQ R-Nylon-6

**Figure 6:**
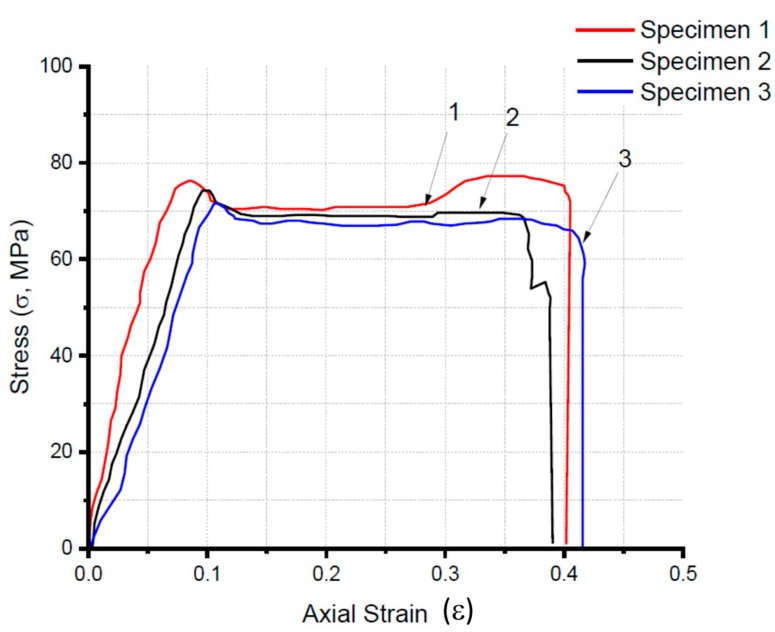
Stress (σ) vs. axial strain (ε) curves of the AQ27000 filaments.

Tensile tests were conducted on five different types of AQ R-Nylons-6, which include AQ27000, AQ27000-B, AQ34000, AQ34000-B, and AQ24000-T filaments. Such tests were performed according to the ASTM D638 standard, using a universal tester Controls 50-C1201/BFR, with maximum capacity 100 kN, maximum vertical clearance 182 mm, horizontal clearance 720 mm, and piston travel of 130 mm. Table 4 shows the results of the tensile tests performed on the AQ27000 filaments in three repetitions, together with the average values (AVG) and the standard deviations (STD) of the examined parameters. The stress–strain curves for all the tested AQ27000 specimens are illustrated in Figure 6. The following section presents a detailed comparison between the mechanical properties of AQ R-Nylon-6, NR R-Nylon-6, and some commercial nylon-based filaments for FDM, which include Nylon PA6 Low Warp by Spectrum [25], Nylon-645 [26] and Nylon-680 [27] by Taulman3D, Nylon-11 (material properties by GoodFellow) [28], and Nylon-12 by Stratasys [29].

#### 3.5.3. Comparison between AQ R-Nylon-6, NR R-Nylon-6, and Commercial Filaments

Table 5 shows a comparison between the mechanical properties of the examined AQ27000 R-Nylon-6 filaments and different nylon materials used in 3D printing processes, which include recycled R-Nylon-6 filaments for FDM, and the commercial nylon filaments listed in the previous section. An overall look at the results presented in Table 5 highlights that AQ27000 R-Nylon-6 filaments exhibit significantly higher mechanical properties than those shown by the NR R-Nylon-6 filaments examined in reference [19], being competitive in terms of mechanical performances with the examined commercial filaments.

Going into the details of the results in Table 5, we observe that the yield strength of the AQ27000 filaments exhibits the following deviations from the analogous strength of the other examined filaments: +745% with respect to pure NR R-Nylon-6, from –8% to +90% over the blended NR R-Nylon-6 described in Section 3.2; +90% over PA6 Spectrum; +113% over Nylon 645; +60% over Nylon 680; +59% over Nylon-11; +138% over Nylon-12. Passing to examine the elongation at break, we observe that the value of such property in AQ27000 is significantly greater than that of pure NR R-Nylon6 filaments, close to that of Nylon 680, Nylon 11, and Nylon 12, and slightly or significantly lower than the elongations at break declared by the manufacturers for the commercial filaments examined in Table 5 (PA6 Spectrum and Nylon 645). Finally, we observe that the Young modulus of AQ27000 filaments exhibits the following deviations when compared with the Young moduli of the other filaments in Table 5: +486% over pure NR R-Nylon-6; from –45% up to −37% over blended NR R-Nylon-6; +9% compared to PA6 Spectrum, +681% over Nylon 645; +9% over Nylon-11; and +26 % over Nylon-12. It is useful to observe that the elongations at break of the AQ27000 filaments in Table 4 are either appreciably larger than, or in line with, those observed, e.g., in the different ABS filaments available in the market (which vary from 2% to 36%, measured through ISO 527–2 [41]). 

Table 6 lists and compares the mechanical properties exhibited by all the examined R-Nylon-6 filaments with those of the AQ27000 filaments. For each analyzed property, the deviation of the average value is observed on three repetitions and is compared with the analogous average value exhibited by the AQ27000 filaments.

The results illustrated in Table 6 shows that AQ27000 (Table 5) filaments have lower tensile strength properties than the AQ27000-B, AQ34000, and AQ34000-B filaments, with more sensitive deviations from the AQ27000-B and AQ34000 filaments. One also observes that AQ27000 filaments have elongation at break not very different from that exhibited by the filaments AQ27000-B, AQ34000, and AQ34000-B. The elongation at break of such filaments is, however, significantly greater than that of the filaments AQ24000-T. It is also worth noting that the Young modulus of the AQ27000 filaments is appreciably lower than that exhibited by the AQ27000-B filaments and slightly greater than those shown by the AQ34000 and AQ34000-B filaments. As for the filaments AQ24000-T, the results in Table 6 show that these filaments exhibit mechanical properties that are lower than those obtained for the AQ27000 filaments. From the analysis of the above data, it can be concluded that the AQ R-Nylon-6 and TiO_2_ mix is disadvantageous in terms of mechanical properties, compared to pure AQ R-Nylon-6. On the contrary, the mix of AQ R-Nylon-6 and ABS is particularly convenient in terms of mechanical properties, when using the AQ R-Nylon-6 with a viscosity number of 2.7 mL/g. Overall, one concludes that the AQ27000-B filaments are the ones with the highest tensile strength and Young modulus, among all the AQ R-Nylon-6 filaments examined in the present work.

### 3.6. Wear Resistance

The wear resistance properties of UNEX AQ27000, AQ27000, and AQ27000-B filaments were studied by conducting experimental investigations through material removal (wear), using a Pin on Disk setup (DUCOM rotary tribometer, with EN32 disk, load range up to 60 N, max speed: 500 rpm, frictional force measurement: 0–20 N, and max wear measurement: 0 to 1200 µm) and Shore D Hardness tests. The wear properties were determined through a pin-on-disk setup featuring a 10 mm diameter pin subject to lateral load, which is in contact with a rotating disk pasted with emery paper of grit size 600 [19]. The operating conditions contemplate a 76 mm track diameter, which operates at a sliding speed of 250 rpm, under a 1 kg load for 15 min, as in the study on NR R-Nylon-6 filaments presented in [19]. The samples’ subject-to-wear tests were manufactured by using a hot-mounting-press technique, to obtain the cylindrical pins to be tested. The results illustrated in Table 7 indicate the highest wear rate (measured through the microns of loss material at the end of the test) in UNEX AQ27000, an intermediate wear level in the extruded material AQ27000, and the minimum wear rate in the extruded AQ27000-B, among the examined materials. It is worth noting that the wear rates in Table 7 are all close to the lowest wear rate (104 μm) measured on pins made with NR R-Nylon 6 FDM materials in [19], under the same test conditions.

**Figure 7:**
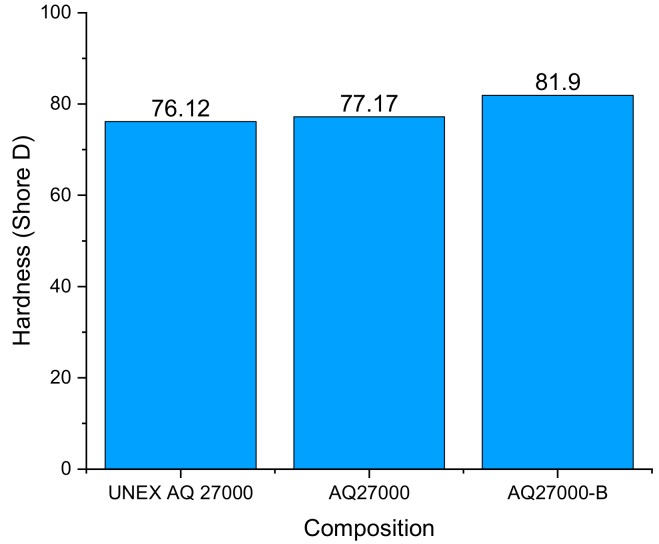
Comparisons between the Shore D Hardness of the examined materials.

A subsequent investigation examined the Shore D Hardness, which was evaluated by using a portable tester (widely used to measure the hardness of rubber or plastic-type materials), according to the standard ISO 868:2003 [41]. Such a tester measures the penetration depth of a tip subjected to constant force. The results of the Shore D hardness tests presented in Figure 7 highlight that the material with the highest Shore D Hardness is AQ27000-B, which also exhibits the highest tensile strength and the highest resistance to wear (Table 7), among the examined AQ R-Nylon-6 materials. The observed values of the Shore D Hardness for the (extruded) AQ filaments are in the range 76–82, and it is worth noting that the values of the same property in commercial ABS filaments range in the same interval [42], which confirms the good wear resistance of the AQ R-Nylon-6 filaments.

### 3.7. 3D Printing Feasibility

We studied the 3D printing feasibility of the AQ27000 and AQ27000-B filaments by using a dual extruder MakerBot Replicator 2X printer (MakerBot^®^ Industries, Hong Kong), with standard specifications of 100 µm resolution, 0.35 mm nozzle diameter, and build platform of 250 × 150 × 150 mm. The 3D printing tests performed on samples with different shapes led us to define the key 3D printing settings of such filaments, which are given in in Table 8. For the sake of comparison, another such study also covered the commercial Spectrum PA6 filament [25]. It is worth noting that Nylon-6 filaments need extrusion temperatures approximately equal to 220–230 °C, as demonstrated by the DSC tests presented in Section 3.4. In the case of the MakerBot Replicator 2X, the optimal melting temperature for the AQ R-Nylon-6 filament was successfully reached at the value of 230 °C for the AQ27000-B filament and the value of 235 °C for the AQ27000 filament. The temperature difference between these filaments, although minimal, is justified by the increased resistance of the AQ27000 filament to the extrusion head [43]. The analyzed AQ filaments are highly hygroscopic, as noted above. For their correct 3D printing it was necessary to preheat the filaments by keeping them in the oven for 8 h at 85 °C, before testing the filaments for 3D printing applications. Once withdrawn from the preheating oven, the filaments were stored in an airtight envelope.

**Figure 8:**
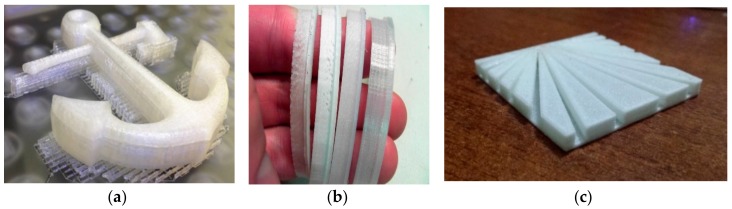
(**a**) Result of the AQ27000 3D printing test on an anchor-shaped sample; (**b**) rings of various shapes obtained by different settings of the slicing parameters with AQ27000 filaments; (**c**) Aquafil logo 3D printed with AQ27000 filaments.

Another issue that may arise when 3D printing with nylon is warping of the layers forming the 3D printed objects, as a consequence of the high temperature gradients occurring between the extruded parts and the environment [44]. Warping tests were run on the anchor-shaped specimens illustrated in Figure 8, through the visual inspection of the curvature of the base of such specimens after their removal from the build platform. We assumed that no appreciable warping occurred if the base of the specimen remained flat after cooling and removal from the platform. The results in Table 9 highlight the good performance of the specimen printed in AQ27000 with a nozzle temperature of 230 °C, which was found optimal for this filament. Differently, the specimen printed with the AQ27000-B filament proved to be more prone to warping effects, which nevertheless could be tackled by setting the plate temperature of the printer at 80 °C and using a nozzle temperature varying in the range 230 and 235°C (cf. Table 10).

After testing the AQ filament filaments, the commercial filament PA6 Low-Warp by Spectrum [25] was subjected to warping tests, using the settings parameters shown in Table 8. Such a filament also performed very well, as no warping or curling issues were observed while printing this material. The same anchor-shaped object was employed for the warping tests on the AQ27000 and AQ27000-B filaments. The surface finishing of the object printed through the PA6 Low-Warp by Spectrum was, however, not perfect, being characterized by appreciable roughness.

## 4. Concluding Remarks

The results presented in this study have demonstrated the high technical potential of the screw-extrusion process as a manufacturing technique for Nylon-6 recycling. The use of ECONYL^®^ grains regenerated from Nylon-6 waste by the Aquafil group [18], alone or in combination with small quantities of ABS and TiO_2_ grains. With regard to the MFI of the examined filaments, it was observed that AQ27000 filaments exhibit significantly higher MFI than all other AQ filaments tested (24.69 g/10 min), except those made for the composite filaments AQ24000-T. The DSC results showed good thermal stability of the AQ27000 material. Furthermore, in the various thermal tests performed at different stages (pre-extrusion and post-extrusion), it was observed that thermal properties of the R-Nylon material remain stable after repeating three cycles of DSC tests. In terms of mechanical performances, it was observed that the R-Nylon AQ27000 filaments exhibit significantly higher tensile yield strength (76.20 MPa) than that shown by the non-regenerated R-Nylon-6 filaments (9.03 MPa), and the examined commercial virgin nylon filaments for FDM (ranging from 32 MPa to 48 MPa, cf. Table 5). It is also worth observing that the elongation at yield (nearly 9%) and that at break (nearly 40%) observed for AQ27000 filaments are either significantly larger than, or in line with, those exhibited by the different ABS filaments available in the market (ranging between 2% to 36% when measured through ISO 527–2) [41,45,46]. The filament with best performances in terms of 3D printing was the pure AQ27000, which was found to be not particularly sensitive to warping effects (cf. Section 3.7). Overall, it can be concluded that AQ R-Nylon granules/filaments have shown great eco-sustainability, excellent mechanical strength, and stable thermal properties after recycling, which makes them feasible to use for various 3D printing applications.

The future direction of the present research may include a comprehensive study of the blending of some of the commonly known reinforcing agents, such as carbon nanotubes and graphene, with Nylon-6, since these substances possess an excellent potential in terms of mechanical resistance, biocompatibility, and various uses in sensors and conductive devices [47,48,49,50,51,52,53]. Blending these substances may lead to the generation of advanced materials to be employed for the additive manufacturing of strong, flexible, and durable structures for a variety of commercial uses [49,54], which need to be investigated via mechanical testing and the microstructure characterization conducted through optical and scanning electron microscope imaging.

## Figures and Tables

**Table 1:** Composition and MFI of NR R-Nylon-6/Al/Al_2_O_3_ composite filaments.

Composition	NR R-Nylon-6	Al	Al_2_O_3_	MFI (g/10 min)
**A**	60	26	14	2.19
**B**	60	28	12	2.25
**C**	60	30	10	2.31

**Table 2:** Evaluating the MFI of AQ27000 filaments out of five repetitions.

Specimen	1st	2nd	3rd	4th	5th	Avg
**MFI (g/10 min)**	29.50	27.30	21.23	21.99	23.44	24.69

**Table 3:** Percentage deviations between the MFI of different composite AQ filaments for FDM and the MFI of pure AQ27000 filaments.

AQ27000-B	AQ34000	AQ34000-B	AQ24000-T
−22.08% (19.24)	−12.07% (21.71)	−17.92% (20.26)	+58.33% (39.09)

Note: The numbers in parentheses indicate the absolute values of the MFI.

**Table 4:** AQ27000 filament tensile test results.

Specimen	1st	2nd	3rd	AVG	STD
Yield strength (MPa)	77.34	76.37	74.9	76.20	1.23
% elongation at Yield	8.18	8.92	10.20	9.10	1.02
Break strength (MPa)	75	69	66	70	4.58
% elongation at break	39.60	38.00	41.59	39.73	1.80
Young modulus (GPa)	1.66	1.64	1.63	1.64	0.01
Speed (mm/min)	50	50	50	50	0

**Table 5:** Comparison of the average values of the mechanical properties of different nylon-based filaments.

Property	AQ27000	STD	Pure NR R-Nylon-6 [19]	Blended NR R-Nylon-6 [20,21,22,23,24]	PA6 Spectrum [25]	Ny-lon 645 [26]	Ny-lon 680 [27]	Ny-lon-11 [28]	Ny-lon-12 [29]
Tensile strength at yield [MPa]	76.20	1.23	9.02	40–83	40	35.77	47.57	48	32
Young modulus [GPa]	1.64	0.01	0.28	2.6–3.0	1.50	0.21	N/A	1.50	1.30
% Elongation at break	40	1.80	14	20–60	250	186	34	35	30

**Table 6:** Percentage deviations between the mechanical properties of different composite filaments.

Property	AQ27000-B	AQ34000	AQ34000-B	AQ24000-T
Yield strength (MPa)	+14.06% (86.91) *	+12.85% (85.99)	+6.57% (81.21)	−26.79% (55.79)
Young’s modulus (GPa)	+42.78% (2.34)	−1.68% (1.61)	−8.72% (1.50)	−53.75% (0.76)
% Elongation at break	−5.89% (37.65)	−0.21% (39.92)	−0.72% (39.71)	−69.32% (12.27)

* The numbers in parentheses indicate the absolute values of the properties.

**Table 7:** Results of the wear tests carried out through a pin-on-disk setup.

Sample	UNEX AQ27000	AQ27000	AQ27000-B
Wear (μm)	102	95	92

**Table 8:** Key print-process management parameters for the AQ27000, AQ27000-B filaments, and a commercial PA6 filament of comparison (Spectrum PA6 Low-Warp [25]).

Parameters	AQ27000	AQ27000-B	Spectrum PA6 [25]
Print plane tilt	No	No	No
Heated printing plane	yes	Yes	Yes
First Layer Weight	0.32 mm	0.36 mm	0.36 mm
First layer extrusion speed	65%	50%	50%
Surrounding temperature	23 °C	23 °C	23 °C
Print speed	50 mm/s	40 mm/s	40 mm/s
Humidity	Absent	Absent	Absent (Dry)
Layer thickness	0.5 mm	0.5 mm	0.5 mm
Nozzle diameter	0.4 mm	0.4 mm	0.4 mm
Filament diameter	1.75 mm	1.75 mm	1.75 mm
Extrusion temperature	235 °C	230 °C	250 °C
Activating cooling fan	after 50 s per layer	after 50 s per layer	after 50 s per layer
cooling fan slow down	after 10 s per layer	after 10 s per layer	after 10 s per layer
Retraction	0.9 mm	0.9 mm	0.9 mm
Retraction speed	15 mm/s	15 mm/s	15 mm/s
Skirt height	1 layer	1 layer	1 layer
Object-skirt Distance	3 mm	3 mm	3 mm
Brim	10 mm	20 mm	10 mm
% Fill (infill)	10%	15%	10%
Infill speed	80 mm/s	80 mm/s	80 mm/s
Perimeter printing speed	60 mm/s	60 mm/s	60 mm/s
Bridge	1.2 cm	1.2 cm	1.2 cm

**Table 9:** Print-test results performed on an anchor sample made with the AQ27000 filament.

	AQ27000						
	Model	Max Size (mm)	T * °C	T ^°C	Raft	Infill Pattern	Result
Trial 1	anchor	50	235	40	No	Hexagonal	No warping
Trial 2	anchor	50	235	60	No	Hexagonal	No warping
Trial 3	anchor	50	235	80	No	Hexagonal	No warping

* Temperature of extruder, ^ Temperature of plate.

**Table 10:** Print-test results performed on an anchor samples made with the AQ27000-B filament.

	AQ27000-B						
	Model	Max Size (mm)	T * °C	T ^°C	Raft	Infill Pattern	Result
Trial 1	anchor	50	230	40	No	Hexagonal	warping
Trial 2	anchor	50	230	60	No	Hexagonal	warping
Trial 3	anchor	50	230	80	No	Hexagonal	No warping
Trial 4	anchor	50	235	80	No	Hexagonal	No warping

* Temperature of extruder; ^ temperature of plate.
